# Editorial: Guidelines From the Central-Eastern European Professional Consensus Statement on Breast Cancer

**DOI:** 10.3389/pore.2022.1610587

**Published:** 2022-07-21

**Authors:** Janina Kulka, Gábor Cserni

**Affiliations:** ^1^ Department of Pathology, Forensic and Insurance Medicine, Semmelweis University, Budapest, Hungary; ^2^ Department of Pathology, Bács-Kiskun County Teaching Hospital, Kecskemét, Hungary; ^3^ Institute of Pathology, Albert Szent-Györgyi Medical Centre, University of Szeged, Szeged, Hungary

**Keywords:** breast cancer, imaging, pathology, surgery, radiotherapy, systemic treatment, follow-up and rehabilitation, psycho-oncology

Multidisciplinary management of breast cancer patients has become standard of care. It has been shown that patients managed by multidisciplinary teams have better disease outcome and better quality of life. In a large retrospective cohort study analyzing outcome data of 13,722 breast cancer patients diagnosed between 1990 and 2000 at an NHS Hospital in Scotland, it was shown that after the introduction of multidisciplinary care, breast cancer mortality was 18% lower compared to hospitals performing traditional care (1).

Guidelines of various disciplines are being updated regularly as developments in the respective fields evolve rapidly. Furthermore, guidelines developed in different continents and countries around the globe adjust their standards not only to recent and widely acknowledged evidence-based developments but take also into account regional/national opportunities, respective quality assurance measures and health care system structure. Wherever professional guidelines are set, their ultimate and uniform aim is to provide and certify the highest possible standards and quality of patient care.

In Hungary, specialists involved in breast cancer patients’ management were the first to recognize the need of a national multidisciplinary document setting basic standards for the respective specialities. In order to develop a consensus document, a multidisciplinary conference was organized in Eger in 1999. The document was approved by the respective professional colleges and from then on the “Consensus Conference Document” became a reference for all medical specialists involved in breast cancer patients’ management. Ten years later, and thereafter on three occasions in the following years, the Consensus Document was updated regularly within the frame of the Kecskemét Consensus Conferences. The last edition of these Consensus Documents was published in 2020 in Hungarian Oncology (2–7). As concerns their development, six panels of experts (one for each document covering the fields of breast screening and imaging; pathology diagnosis; surgery; radiotherapy; systemic treatment; follow-up, rehabilitation and psycho-oncology) were invited to draft a document on the basis of previous editions and novel changes in practices and recommendations around the world, and make it available for public consultation 1–2 months before the Consensus Conference. Professionals, including members of the other professional panels were invited to comment the recommendations in the documents in writing or at the Consensus Conference, and the texts were amended according to the relevant comments received prior to acceptance by the panel and publication.

Since Central European countries share many similarities including the incidence of breast cancer, health care structure and both financial and instrumental opportunities of cancer treatment in general and in breast cancer treatment in particular, it was a logical step to share and discuss the most recent Consensus Document in a wider circle of Central and Eastern European countries’ specialists. The initiative came to birth with the help of a ministerial support and the enthusiastic work of a leading breast oncoplastic surgeon, Dr. Zoltán Mátrai, founding member of the Central-Eastern European Breast Cancer Surgical Consortium (CEEBCSC) and first president of the Central and Eastern European Academy of Oncology (CEEAO).

The development of the final version of the texts published in this Issue of Pathology & Oncology Research followed a methodology similar to the formulation of the Hungarian Consensus Documents; i.e., the Hungarian documents (2–7) were translated, updated where deemed necessary, circulated to institutions involved in the CEEAO and their networks, modified according to comments, made available for public discussion prior to a hybrid Consensus Conference held in Visegrád, Hungary on 5–7 November 2021. This resulted in the final adjustment of the recommendations formulated through partners in the CEEAO following the international discussions, and the production of a set of up-to-date guideline-type documents that reflect the gold standard of breast cancer patients’ management in our region. Meanwhile it has to be emphasized that all documents included in the Issue incorporated the basic standards of the most recent European and American guidelines.

The set of documents published in this issue of Pathology & Oncology Research (8–13) comprises the guidelines for the major specialties involved in breast cancer management: radiology and nuclear medicine for imaging, pathology, surgery, medical- and radiation oncology and rehabilitation, including psycho-oncology. Compared to the 2020 texts of the Hungarian recommendations, the pathology (9), surgery (10) and medical oncology (12) texts have been substantially updated, the radiation oncology text had minor modification, whereas the other two texts had no mentionable changes.

In the first document (8), standards of multimodality imaging from mammography to isotope localization techniques, imaging follow-up of cancer patients and technical requirements of the instruments are described in line with the most recent specific professional guidelines.

The second document on pathology workup and reporting (9) includes guidance from processing of the material to its reporting and content of the report, an updates also cover the use of digital- and molecular pathology methods acceptable as standards.

The third text (10) includes the modern approach of oncoplastic surgery and suggests to treat breast cancer patients in centers where this modality is available.

The fourth document (11) summarizes the evidence-based modern methods and technical requirements of radiation oncology used in the adjuvant treatment of breast cancer patients.

The fifth document on medical oncology (12) describes in detail state-of-the art medical treatment of breast cancer patients, including the most recent opportunities provided by immune therapies and therapies based on the results of multigene molecular testing of tumors.

The sixth document gives guidance for follow up, rehabilitation and psycho-oncology (13) and is an important chapter in the Issue which was accepted by the International Consensus Committee. We believe that publishing these guidelines will help medical teams to achieve high standards of breast cancer patients’ management and breast cancer patients to have better outcome of their disease.
